# IIb or not IIb? Regulation of myosin heavy chain gene expression in mice and men

**DOI:** 10.1186/2044-5040-1-5

**Published:** 2011-02-01

**Authors:** Brooke C Harrison, David L Allen, Leslie A Leinwand

**Affiliations:** 1 Department of Molecular, Cellular, and Developmental Biology, University of Colorado at Boulder, Boulder, CO 80309, USA; 2 Department of Integrative Physiology, University of Colorado at Boulder, Boulder, CO 80309, USA

## Abstract

**Background:**

While the myosin heavy chain IIb isoform (MyHC-IIb) is the predominant motor protein in most skeletal muscles of rats and mice, the messenger RNA (mRNA) for this isoform is only expressed in a very small subset of specialized muscles in adult large mammals, including humans.

**Results:**

We identify the DNA sequences limiting MyHC-IIb expression in humans and explore the activation of this gene in human skeletal muscle. We demonstrate that the transcriptional activity of ~1.0 kb of the human MyHC-IIb promoter is greatly reduced compared to that of the corresponding mouse sequence in both mouse and human myotubes *in vitro *and show that nucleotide differences that eliminate binding sites for myocyte enhancer factor 2 (MEF2) and serum response factor (SRF) account for this difference. Despite these differences, we show that MyHC-IIb mRNA is expressed in fetal human muscle cells and that MyHC-IIb mRNA is significantly up-regulated in the skeletal muscle of Duchene muscular dystrophy patients.

**Conclusions:**

These data identify the genetic basis for a key phenotypic difference between the muscles of large and small mammals, and demonstrate that mRNA expression of the MyHC-IIb gene can be re-activated in human limb muscle undergoing profound degeneration/regeneration.

## Background

At the level of the sarcomere, one of the greatest differences in gene expression between mouse and human skeletal muscle is found within the myosin heavy chain (MyHC) gene family. This gene family consists of one cardiac specific isoform (MyHC-α), two developmental isoforms (MyHC-embryonic and MyHC-perinatal), one specialized eye muscle isoform (MyHC-extraocular), one isoform that is expressed in both cardiac and skeletal muscle (MyHC-β) and three skeletal-specific isoforms (MyHC-IIa, MyHC-IId/x and MyHC-IIb) [1,2]. The expression of a particular MyHC isoform within an individual muscle fibre confers specific morphological, enzymatic and functional effects on that muscle fibre such that MyHC-β-expressing fibres are typically smaller, slower contracting fibres rich in the enzymes of oxidative metabolism while MyHC-IIb-expressing fibres are typically larger, faster contracting fibres dependent on glycolytic pathways of energy generation [3,4].

Of the adult skeletal isoforms, MyHC-β, -IIa and -IIx are each expressed to varying degrees in both mouse and human skeletal muscle. However, although MyHC-IIb is highly expressed at both the messenger RNA (mRNA) and protein level in murine skeletal muscle, evidence to date suggests that this isoform is effectively only expressed at the mRNA level in a very small subset of specialized muscles in the adult human [5-10]. As mentioned above, MyHC-IIb expression is typically associated with high forces of contraction combined with rapid contractile characteristics and it has been suggested that the contractile characteristics of MyHC-IIb may be incompatible with the biomechanical constraints of larger muscles [7,11]. Despite this potential incompatibility, it has also been suggested that the MyHC-IIb gene might be a target for 'gene doping' manipulations designed to improve human athletic performance [12,13]. Given that the human MyHC-IIb gene is intact, highly conserved with the mouse MyHC-IIb gene and capable of producing functional, enzymatically active myosin if expressed as recombinant protein [1,2,9,14], we sought to elucidate the molecular mechanism responsible for the species difference in MyHC-IIb expression between the mouse and the human.

## Results

### The human MyHC-IIb promoter has reduced transcriptional activity compared to the corresponding mouse sequence

**Figure 1:**
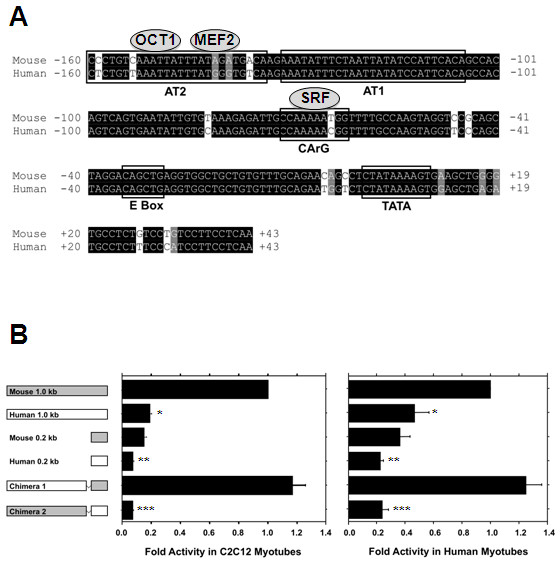
**Myosin heavy chain IIb (MyHC-IIb) promoter sequence alignment and activity in mouse and human muscle cells**. (A) For the proximal 0.2 kb of the mouse and human MyHC-IIb promoters, the sequence identity was 94%. Both sequences contain a conserved E-Box and AT rich region (AT1), along with a consensus TATA box. However, the AT2 and CArG box regions contain sequence differences predicted to affect myocyte enhancer factor 2 (MEF2) and serum response factor (SRF) binding to the human sequence. (B) Activities of the 1.0 kb and 0.2 kb human constructs were significantly reduced compared to the corresponding mouse sequences in both the mouse and human cells. A chimeric construct containing the proximal human 0.2 kb region linked to the distal mouse 0.8 kb region (Chimera 2) showed reduced activity compared to the opposing construct (Chimera 1). Values (mean ± standard error of mean) represent the result of at least three duplicate experiments and are expressed as fold-activity relative to the mouse 1.0 kb MyHC-IIb reporter plasmid. *P < 0.01 Human 1.0 kb versus Mouse 1.0 kb; **P < 0.05 Human 0.2 kb versus Mouse 0.2 kb; ***P < 0.01 Chimera 2 versus Chimera 1.

Given the high identity of the mouse and human MyHC isoform gene clusters and that ~1.0 kb of the proximal mouse MyHC-IIb promoter region is sufficient to confer both muscle and fibre-type specificity [1,15-18], we began our investigation by aligning ~1.0 kb of the mouse and human MyHC-IIb promoter regions. This sequence alignment revealed 79% identity between the two species across the ~1.0 kb regions (data not shown). The identity between the mouse and human sequences for the first 0.2 kb upstream from the TATA box was 94% and an analysis of potential transcription factor binding sites revealed a similar pattern of muscle-specific transcription factor binding sites (Figure 1a). Importantly, both the mouse and human sequences contain a consensus E-box site, two AT rich regions previously shown to bind myocyte enhancer factor 2 (MEF2) [15,16,19,20] and a CArG box motif [16] (Figure 1a).

Based on these results, we performed transient transfection studies using the mouse and human 1.0 kb upstream regions in both mouse (C2C12) and human (primary fetal myotubes) muscle cells. These experiments revealed that the 1.0 kb human MyHC-IIb promoter was approximately 20% as transcriptionally active as the same length of mouse IIb sequence in C2C12 cells and ~40% as active in human myotubes (Figure 1b). Deletion constructs containing only the proximal 0.2 kb of the promoter region for both species exhibited diminished activity compared with that of the 1.0 kb constructs but the activity of the human 0.2 kb construct was still significantly lower than that of the corresponding mouse construct in both mouse and human cells (Figure 1b). In addition, the activity of a chimeric promoter construct containing the distal human 0.8 kb linked to the proximal mouse 0.2 kb (Chimera 1) was as high as, or higher than, that of the 1.0 kb mouse construct (Figure 1b), while activity of a construct containing the distal mouse 0.8 kb linked to the proximal human 0.2 kb (Chimera 2) was similar to that of the human 0.2 kb region alone (Figure 1b). These results indicated that much, if not all, of the reduced activity of the human MyHC-IIb promoter region could be attributed to the proximal 0.2 kb region.

### The reduced transcriptional activity of the human MyHC-IIb promoter is due to diminished binding of MEF2 and serum response factor (SRF)

**Figure 2:**
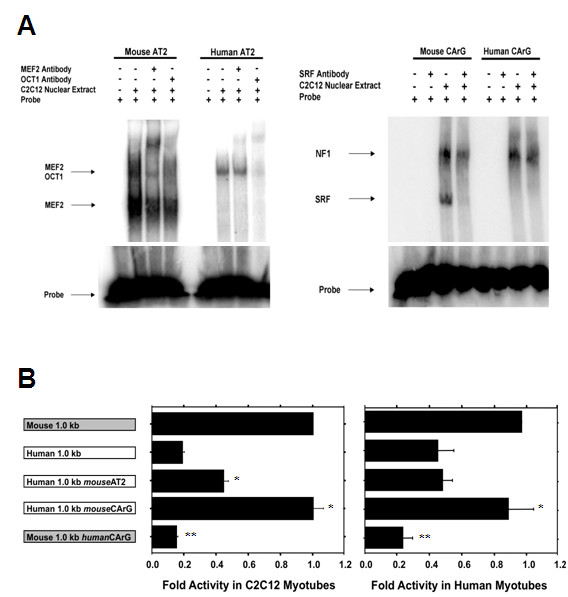
**Electrophoretic mobility shift assays (EMSA) and transient transfection of site-directed mutagenesis constructs**. (A) EMSA results using radiolabelled probes for the mouse and human AT2 and CArG box regions with C2C12 nuclear extract. The mouse AT2 region showed strong binding of a protein that was supershifted by a myocyte enhancer factor 2 (MEF2)-specific antibody. The human probe did not bind this protein, but did bind a protein that was supershifted by an octamer binding transcription factor 1 (OCT1)-specific antibody. The mouse CArG probe demonstrated strong binding to a protein that was supershifted by a serum response factor (SRF)-specific antibody while the human probe did not bind this protein. (B) Reporter plasmid transfection results indicated that targeted mutagenesis of the human CArG box motif (Human 1.0 kb *mouse*CArG) increased activity of the human MyHC-IIb promoter construct ~fivefold in mouse cells and ~twofold in human cells, resulting in activity equal to that of the full-length mouse construct. Conversely, replacing the mouse CArG box sequence with the corresponding human sequence significantly reduced activity of the mutated mouse construct (Mouse 1.0 kb *human*CArG) in both mouse and human cells. Mutagenesis of the human AT2 region (Human 1.0 kb *mouse*AT2) increased activity ~twofold in mouse cells but had no effect in human cells. Values (mean ± standard error or mean) represent the result of at least three duplicate experiments and are expressed as fold-activity relative to the mouse 1.0 kb MyHC-IIb reporter plasmid. *P < 0.05 Human 1.0 kb *mouse*AT2 or *mouse*CArG versus Human 1.0 kb; **P < 0.05 Mouse 1.0 kb *human*CArG versus Mouse 1.0 kb.

As described above, an *in silico *analysis of the mouse and human MyHC-IIb promoter regions revealed distinct patterns of muscle-specific transcription factor binding sites. More specifically, the proximal mouse MyHC-IIb promoter contains two AT rich regions at -104/-133 (AT1) and -141/-158 (AT2) upstream of the TATA box previously shown to bind to MEF2 and octamer binding transcription factor 1 (OCT1) (Figure 1a) [15,16]. Although AT1 is 100% conserved in the human, the AT2 region has five nucleotides that differ between the two species, including a base difference (position -142) in a region known to bind MEF2 (Figure 1a) [15,16]. Electrophoretic mobility shift assays (EMSA), using probes for the mouse and human AT2 regions, indicated that the mouse AT2 region demonstrated strong binding to a protein present in mouse myotube nuclear extract that was supershifted by an MEF2-specific antibody (Figure 2a). This protein binding was not observed with the human AT2 sequence, although the human AT2 region did bind a protein from the mouse myotube nuclear extract that was supershifted by an OCT1-specific antibody (Figure 2a). The EMSA analysis of the AT2 region using human myotube nuclear extract revealed a similar pattern (data not shown), suggesting that the observed differences in MEF2 binding were not due to differences in binding affinity between mouse and human MEF2. These results indicate that the mouse AT2 region is strongly bound by MEF2 and OCT1, while the human AT2 region binds OCT1 but not MEF2.

As mentioned above, the proximal 0.2 kb region of the mouse promoter also contains a consensus CArG box motif (CC(A/T)_6_GG) at position -62 (Figure 1a) [15]. The human sequence, however, contains a single nucleotide difference that results in a non-consensus CArG-*like *motif (CCAAAAAcGG). EMSA, using oligonucleotide probes containing either the mouse or the human CArG box regions, showed specific binding by the mouse probe to a protein present in C2C12 myotube nuclear extracts that was supershifted by an SRF-specific antibody, while the human probe did not bind this protein (Figure 2a). Both the mouse and human CArG probes showed similar binding to a second protein, believed to be nuclear factor 1 (NF1), as this protein has been previously reported to bind this region of the mouse MyHC-IIb promoter [15]. The EMSA analysis using human myotube nuclear extract also revealed binding to the mouse promoter of a protein recognized by an SRF-specific antibody (data not shown), further confirming that this CArG box-mediated effect on IIb expression is a cis-effect and is not due to a deficiency of SRF protein in human cells. Together, these results indicate that the CArG motif of the mouse MyHC-IIb promoter region binds the SRF protein and that a single base difference within the human sequence greatly reduces, or abolishes, SRF binding.

Given these binding differences between the AT2 and CArG-box regions of the mouse and the human MyHC-IIb promoters, we used targeted mutagenesis to create human MyHC-IIb promoter constructs containing either the mouse AT2 or CArG-box regions. Mutagenesis of the human AT2 region modestly increased the activity of this construct (human 1.0 kb *mouse*AT2) in C2C12 myotubes and had no effect in human myotubes (Figure 2b). However, mutagenesis of the human CArG*-like *motif (CCAAAAAcGG) to the mouse sequence (CCAAAAATGG; human 1.0 kb *mouse*CArG), increased activity fivefold in C2C12 myotubes and twofold in human myotubes, resulting in activity equal to that of the wild type 1.0 kb mouse construct (Figure 2b). Conversely, mutating the mouse CArG to the human sequence significantly decreased activity of this construct (mouse 1.0 kb *human*CArG) and resulted in activity comparable to that of the wild type 1.0 kb human construct (Figure 2b). These results suggest that the CArG box motif in the mouse MyHC-IIb promoter region is both necessary and sufficient for its relatively higher-level activity.

### The human MyHC-IIb promoter region is responsive to stimuli and MyHC-IIb messenger RNA (mRNA) is expressed in fetal human muscle cells and Duchene muscular dystrophy biopsy samples

**Figure 3:**
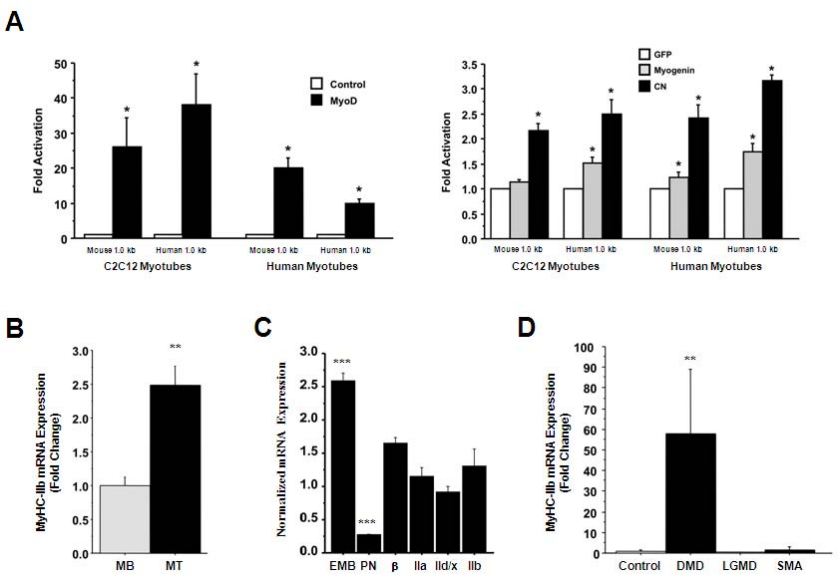
**Myosin heavy chain IIb isoform (MyHC-IIb) promoter responsiveness and messenger RNA (mRNA) expression in human muscle cells**. (A) The mouse and human 1.0 kb MyHC-IIb promoter regions were equally responsive to myogenic regulatory factor (MyoD, myogenin and calcineurin (CN)) over-expression in both mouse and human cells. (B) MyHC-IIb mRNA expression was significantly greater in primary fetal human myotubes (MT) compared to confluent myoblasts (MB). (C) In differentiated (day 5) human myotubes, the level of MyHC-IIb mRNA expression was comparable to that seen for MyHC-β, -IIa and IId/x. Overall, MyHC-Embryonic demonstrated the highest relative expression and MyHC-Perinatal the lowest. (D) MyHC-IIb mRNA expression was significantly increased in Duchene muscular dystrophy (DMD) biopsy samples (*n *= 7) compared to healthy control samples (*n *= 7), limb-girdle muscular dystrophy (LGMD) samples (*n *= 4) or spinal muscular atrophy (SMA) samples (*n *= 2). Values represent mean ± standard error of mean and are the result of at least three duplicate experiments. *P < 0.05 versus control or green fluorescent protein (GFP); **P < 0.01 versus confluent myoblasts or control, ***P < 0.05 versus MyHC-IIb.

As the human MyHC-IIb gene has remained under evolutionary constraint and the promoter is not functionally inactive (albeit transcriptionally repressed relative to the mouse), we asked whether the human MyHC-IIb promoter was capable of responding to other transcription factors known to influence MyHC gene activity. In order to test this, we analysed the affects of over-expression of MyoD, myogenin or calcineurin on the activity of the mouse and human 1.0 kb constructs. In each case, the relative fold-activation in response to these factors was similar for both the mouse and human promoter constructs, although the absolute levels of activity remained lower for the human construct compared to the mouse construct (Figure 3a and data not shown). Of the three transcription factors analysed, MyoD over-expression had the greatest effect on MyHC-IIb promoter construct activity (Figure 3a).

Having demonstrated that the human IIb promoter is capable of responding to factors known to influence MyHC gene expression, we then wished to see if we could find evidence of MyHC-IIb mRNA expression in human muscle cells. In fact, MyHC-IIb mRNA was significantly increased with differentiation in primary fetal human myotubes (Figure 3b) and, after 5 days of differentiation, the level of MyHC-IIb mRNA expression was comparable to that of the other adult skeletal MyHC isoforms (MyHC-β, -IIa and -IId/x; Figure 3c). Given that muscle fibre regeneration is characterized by a re-capitulation of developmental programmes of gene expression that includes re-expression of the developmental myosin heavy chain isoforms and myogenic regulatory factors [19-22], we also wished to see if there was any evidence of MyHC-IIb expression in post-natal human muscle undergoing regeneration in response to muscular disease. Intriguingly, we observed a significant increase in MyHC-IIb mRNA in Duchene muscular dystrophy (DMD) leg muscle biopsy samples compared with leg muscle biopsy samples from healthy control subjects or patients with muscular dystrophies not characterized by rounds of degeneration and regeneration [limb-girdle muscular dystrophy (LGMD) or spinal muscular atrophy (SMA); Figure 3d]. Overall, these results indicate that, although the basal transcriptional activity of the human MyHC-IIb promoter is reduced relative to that of the mouse promoter, the human promoter is responsive to myogenic factors and MyHC-IIb mRNA is expressed *in vitro *in developing fetal myotubes and *in vivo *with severe muscle fibre degeneration/regeneration.

## Discussion

MyHC is the molecular motor for muscle contraction and distinct expression patterns of expression for the individual MyHC isoforms, each with its own unique functional properties, allows for a broad range of muscle fibre contractile characteristics along with the elegant adaptability of skeletal muscle to alterations in activation state. In addition to spatial and temporal variations in MyHC gene expression, there are also species-dependent variations in MyHC gene expression. This species-to-species variation in MyHC isoform expression is, perhaps, most notable for MyHC-IIb, which is the predominant MyHC isoform expressed in most small rodent muscles yet effectively not expressed in the vast majority of adult human skeletal muscle [6,7,10]. Although it has been hypothesized that the contractile characteristics of MyHC-IIb are somehow incompatible with human (and other large mammal) muscle architecture, the underlying molecular reason(s) why the gene is not expressed in the vast majority of human skeletal muscle remains unknown. Given that the human MyHC-IIb gene is intact and highly conserved with the corresponding mouse gene, our intent was to elucidate the molecular mechanism for this marked species difference in gene expression.

Our findings show that, despite highly conserved upstream regulatory regions, the mouse and human MyHC-IIb genes exhibit dramatically different basal transcriptional activities in both mouse and human muscle cells (Figure 1). *In silico *analysis combined with deletional and site-directed mutagenic analyses revealed that the low transcriptional activity of the human MyHC-IIb gene is largely the result of reduced MEF2 and SRF binding within the proximal promoter region (Figure 2). Remarkably, much of the difference in transcriptional activity between the mouse and human MyHC-IIb genes can be attributed to a single nucleotide difference between the two species that effectively eliminates SRF binding to the proximal human MyHC-IIb promoter region (Figure 2), although we can not rule out the possibility of additional regulatory regions outside of the ~1.0 kb used in these studies that may also contribute to the low *in vivo *expression of the human gene.

In both mouse and human cells, the human MyHC-IIb promoter region was responsive to MyoD, myogenin and calcineurin over-expression. These data indicate that, while SRF binding to the CArG motif (and, to a lesser extent, MEF2 binding to the AT2 region) is critical for establishing basal expression, transcriptional activity of the human MyHC-IIb promoter can be induced by other key myogenic regulatory factors. With respect to calcineurin, it is likely that this regulation is modulated via one or both of the consensus nuclear factor of activated T cells (NFAT) binding motifs found in the proximal ~1.0 kb human MyHC-IIb promoter region (-608/-617 and -682/-690).

However, despite reduced basal activity our findings indicate that MyHC-IIb mRNA is expressed in primary fetal human muscle cells *in vitro *(Figure 3). These data led us to hypothesize that the human MyHC-IIb promoter region may be active in developing human musculature and in human tissue undergoing muscle fibre regeneration, as this process is characterized by fetal patterns of gene expression including the expression of developmental MyHC isoforms and potent myogenic regulatory factors such as MyoD. In fact, investigation of MyHC expression in human muscular disorders characterized by severe muscle fibre degeneration/regeneration demonstrated a significant up-regulation of MyHC-IIb mRNA in DMD patients but not in patients with muscle disorders characterized by less severe muscle degeneration/regeneration (LGMD or SMA; Figure 3). Given that the human MyHC-IIb promoter region responds robustly to MyoD over-expression and that MyoD is strongly induced in DMD [19], this observed increase in MyHC-IIb mRNA expression in the DMD samples is likely attributed to MyoD-mediated trans-activation of the human gene.

Although this evidence of increased MyHC-IIb mRNA expression in fetal human myotubes and in DMD skeletal muscle is intriguing, we have not been able to find compelling evidence of MyHC-IIb protein expression in these same samples. Immunohistochemical analyses using the MyHC-IIb-specific antibody BF-F3 [23] appeared to indicate an increased immunoreactivity in DMD samples as compared to control samples but the data were inconsistent. Similarly, while mass spectroscopy detected a small number of peptides attributed to MyHC-IIb in both control and DMD protein isolates, the data did not provide a quantitative assessment of the relative degree of MyHC-IIb protein expression in the two conditions. Our inability to find evidence of MyHC-IIb protein, despite significant activation of the mRNA transcript, parallels the results of Horton *et al*., who also demonstrated MyHC-IIb mRNA expression in the apparent absence of MyHC-IIb protein in the human masseter [9]. Our data, combined with these previous data, indicate that MyHC-IIb expression in the human may be subject to both transcriptional and post-transcriptional control.

## Conclusions

Overall, our data provide the first in-depth analysis of the factors controlling transcriptional activation of the human MyHC-IIb gene and identify key genomic sequence differences that apparently account for the reduced transcriptional activity of the human MyHC-IIb gene relative to that of the mouse gene. We also provide the first evidence that MyHC-IIb mRNA is expressed in fetal human myotubes and is up-regulated in diseased human skeletal muscle undergoing severe muscle fibre degeneration/regeneration. Although these data are intriguing, any speculation on the possible functional significance of MyHC-IIb expression during human development or in the regeneration of human skeletal muscle is limited without any compelling evidence of MyHC-IIb protein expression across these conditions. Further studies across the developmental time points and muscle groups, along with a more extensive analysis of skeletal muscle disease states, may help to determine if, in fact, MyHC-IIb protein is expressed in the human and if the expression of this myosin has any functional consequences.

## Methods

### Cloning and cell culture

The human MyHC-IIb promoter region was identified by a pairwise BLAST search using the previously identified mouse 1.0 kb MyHC-IIb promoter region and human chromosome 17, clone hRPK.799_N_11 sequence (AC005323). Mouse and human MyHC-IIb promoter regions were then amplified by polymerase chain reaction (PCR) and cloned into a *Firefly *luciferase reporter plasmid (VR1255, Vical, CA, USA) at MSCI and SACII. Deletion and chimeric constructs were created using PCR and inverse-PCR with the 1.0 kb mouse and human plasmids as template DNA. Site-directed mutagenesis was performed using inverse-PCR. C2C12 mouse myoblasts were grown in Dulbecco's modified eagle medium (DMEM)(Invitrogen, CA, USA) with 20% fetal bovine serum (FBS). Primary fetal human myoblasts were grown in DMEM with 15% fetal bovine serum (FBS) and 0.05% chick embryo extract (Invitrogen). Both mouse and human myoblasts were differentiated in DMEM containing 1% horse serum (HS). MyHC-IIb promoter plasmids were transfected into C2C12 mouse myoblasts at 85% to 95% confluency using Lipofectamine 2000 (Invitrogen) according to manufacturer's instructions. Human myoblasts were transfected at 85% to 95% confluency using GeneJuice (Novagen, WI, USA) according to manufacturer's instructions. All transfections included a thymidine kinase-*Renilla *luciferase construct (Promega, WI, USA) at a 4:1 ratio (IIb promoter construct: pRLTK) as an internal control. For MyoD over-expression analysis, mouse and human ~1.0 kb IIb promoter plasmids were co-transfected with either a control vector (cytomegalovirus-β-galactosidase) or a MyoD expression vector at a 1:1 ratio (IIb plasmid: control vector or MyoD vector). For myogenin and calcineurin over-expression analysis, cells were transfected at 70% to 80% confluency, as described above, and then infected the following day with either a green fluorescent protein (control), myogenin, or calcineurin-expressing adenoviral vector at a multiplicity of infection (MOI) of 50. Following transfections, confluent cells were differentiated in DMEM with 1% HS for 3 to 5 days at which time, cells were lysed in 1x Passive Lysis Buffer (Promega) and assayed for relative luciferase levels (Firefly/Renilla) using a dual luciferase reporter assay system (Promega) according to manufacturer's instructions.

### Electrophoretic mobility shift assays

Double-stranded, ^32^P radiolabelled probes (100,000 to 150,000 cpm) containing the mouse/human AT2 regions (Mouse AT2 sense 5'-TAGATCATCCCCTGTCAAATT ATTTATAGATGA-3', antisense 5'-TTGTCATCTATAAATAATTTGACAGGGGATGAT-3'; Human AT2 sense 5'-TAGATCATCCTCTGTTAAATTATTTATGGGTGT-3', antisense 5'-TTGACACCCATAAATAATTTAACAGAGGATGAT-3') and the mouse/human CArG box regions (Mouse CArG sense 5'-GTGTAAAGAGATTGCCAAAAATGGTTTTGCCAAGTA-3', antisense 5'-ACCTACTTGGCAAAACCATTTTTGGCAATCTCTTTA-3', and Human CArG sense 5'-GTGCAAAGAGATTGCCAAAAACGGTTTTGCCAAGTA-3', antisense 5'-ACCTACTTGGCAAAACCGTTTTTGGCAATCTCTTTG-3') were incubated with 8-12 μg of nuclear extract isolated from either C2C12 mouse myotubes or primary human myotubes in the presence or absence of antibodies specific to MEF2, OCT1, or SRF (Santa Cruz Biotechnology, Inc, CA, USA). Following incubation, samples were run on a 4% acrylamide binding gel at 300 V for 1 hour and visualized with a Storm PhosphorImager (Molecular Dynamics, CA, USA).

### Human MyHC-IIb mRNA expression analysis

DMD, LGMD and SMA biopsy samples were kindly provided by Dr E Hoffman. Control muscle biopsy samples were surgically isolated from the vastus medialis in sedentary college-age men and were collected in Boulder, Colorado, USA. All protocols were approved by the Human Research Committee, University of Colorado at Boulder, Boulder, Colorado, USA. Total RNA was isolated from human myotubes or biopsy samples using TRI Reagent (Molecular Research Center, Inc, Ohio, USA) according to manufacturer's instructions and stored at -80°C. Following DNase treatment (Turbo DNase, Applied Biosystems/Ambion, TX, USA), first-strand cDNA was synthesized using reverse transcriptase (SuperScript II, Invitrogen), random hexamers (Invitrogen) and 2 μg of total RNA according to manufacturer's instructions. Quantitative real-time PCR was performed using the absolute quantification (standard curve) method with gene specific primers and SYBR Green (Applied Biosystems 7500 System, CA, USA). The following MyHC isoform-specific primers were used: MyHC-Embryonic sense 5'-CCTTCTGGAGCAGGACAGAA-3', antisense 5'-CAAAGCAAAGTTTATTGCATGTG-3'; MyHC-Perinatal sense 5'-TAAACACACCTGCCTGATGC-3', antisense 5'-TCAGCTTTAACAGGAAAATAAACG-3'; MyHC-β sense 5'-TGCCACATCTTGATCTGCTC-3'; antisense 5'-CTCGGCTTCAAGGAAAATTG-3'; MyHC-IIa sense 5'-CTGATGCCATGGAATGACTG-3', antisense 5'-CCCTATGCTTTATTTCCTTTGC-3'; MyHC-IId/x sense 5'-ACATTGCTGAGTCCCAGGTC-3', antisense 5'-TCTTTGGTCACCTTTCAGCA-3'; MyHC-IIb sense 5'-CAAGAGACAAGCTGAAGAGGCT -3', antisense 5'- GATATACAGGACAGTGACAAAGAACT -3'. Due to the high identity across MyHC isoforms, the MyHC-IIb PCR product was sequenced and confirmed to be MyHC-IIb. Alpha-skeletal actin (sense 5'-CGACATCAGGAAGGACCTGTATGCC-3'; antisense 5'-GGCCTCGTCGTACTCCTGCTTGG-3') or ribosomal 18 S (sense 5'-GCCGCTAGAGGTGAAATTCTT-3'; antisense 5'-CTTTCGCTCTGGTCCGTCTT-3') was used as the normalizing gene (18 S for fetal myoblast cell culture experiments; α-skeletal actin for biopsy experiments).

### Statistical analysis

All data are presented as mean ± standard error of mean. Data were analysed for statistical significance using Statview™ 5.0 statistical software (SAS Institute, NC, USA). Results were analysed with ANOVA combined with the Fishers paired least significant difference post hoc test, with statistical significance set at *P *< 0.05.

## Abbreviations

DMD: Duchene muscular dystrophy; EMSA: electrophoretic mobility shift assay; FBS: fetal bovine serum; LGMD: limb-girdle muscular dystrophy; MEF2: myocyte enhancer factor 2; mRNA: messenger RNA; MyHC-IIb: myosin heavy chain IIb; PCR: polymerase chain reaction; SMA: spinal muscular dystrophy; SRF: serum response factor.

## Competing interests

The authors declare that they have no competing interests.

## Authors' contributions

BCH designed the study, carried out all experiments and drafted the manuscript. DLA assisted with the cloning and EMSA studies, participated in the study design and helped draft the manuscript. LAL participated in study design and helped draft the manuscript. All authors read and approved the final manuscript.
